# Dose-Related Reduction in Hippocampal Neuronal Populations in Fetal Alcohol Exposed Vervet Monkeys

**DOI:** 10.3390/brainsci12091117

**Published:** 2022-08-23

**Authors:** Mark W. Burke, Hocine Slimani, Maurice Ptito, Frank R. Ervin, Roberta M. Palmour

**Affiliations:** 1 Department of Physiology and Biophysics, Howard University, Washington, DC 20059, USA; 2 School of Optometry and Department of Physiology, Université de Montréal, Montréal, QC H3C 3J7, Canada; 3 Department of Neuroscience, University of Copenhagen, DK-2200 Copenhagen, Denmark; 4 Behavioural Science Foundation, St. Kitts, Saint Kitts and Nevis; 5 Department of Psychiatry, Faculty of Medicine, McGill University, Montréal, QC H3A 1A1, Canada; 6 Departments of Human Genetics and Psychiatry, Faculty of Medicine, McGill University, Montréal, QC H3A 1A1, Canada

**Keywords:** fetal alcohol exposure, hippocampus, immature neurons, stereology

## Abstract

Fetal alcohol spectrum disorder (FASD) is a chronic debilitating condition resulting in behavioral and intellectual impairments and is considered the most prevalent form of preventable mental retardation in the industrialized world. We previously reported that 2-year-old offspring of vervet monkey (*Chlorocebus sabeus*) dams drinking, on average, 2.3 ± 0.49 g ethanol per Kg maternal body weight 4 days per week during the last third of pregnancy had significantly lower numbers of CA1 (−51.6%), CA2 (−51.2%) and CA3 (−42.8%) hippocampal neurons, as compared to age-matched sucrose controls. Fetal alcohol-exposed (FAE) offspring also showed significantly lower volumes for these structures at 2 years of age. In the present study, we examined these same parameters in 12 FAE offspring with a similar average but a larger range of ethanol exposures (1.01–2.98 g/Kg/day; total ethanol exposure 24–158 g/Kg). Design-based stereology was performed on cresyl violet-stained and doublecortin (DCX)-immunostained sections of the hippocampus. We report here significant neuronal deficits in the hippocampus with a significant negative correlation between daily dose and neuronal population in CA1 (*r*^2^ = 0.486), CA2 (*r*^2^ = 0.492), and CA3 (*r*^2^ = 0.469). There were also significant correlations between DCX population in the dentate gyrus and daily dose (*r*^2^ = 0.560). Both correlations were consistent with linear dose-response models. This study illustrates that neuroanatomical sequelae of fetal ethanol exposure are dose-responsive and suggests that there may be a threshold for this effect.

## 1. Introduction

Exposure to ethanol in utero is recognized as a leading cause of preventable developmental disorder. The clinical continuum of fetal alcohol spectrum disorder (FASD) ranges from craniofacial dysmorphology and mental retardation to the much more common spectrum of developmental learning and behavior disorders. Many of these deficits may not be apparent until the educational years [1] and are typically not recognized as a result of fetal alcohol exposure (FAE). The prevalence of FASD has been estimated to be between 1–4% of live births [2,3], but up to 13% of pregnant women reportedly drink moderate amounts of ethanol during pregnancy [2,4,5], with potentially much higher prevalence in select populations [3,6]. Failure to diagnose less severe cases of FASD in the clinical setting [1,7] may perpetuate fetal alcohol exposure, and this in turn may contribute to a public misconception that moderate prenatal alcohol exposure, particularly during the last trimester, has relatively little impact on the developing fetus [8]. Amongst Fellows of the American College of Obstetricians and Gynecologists (Ob/Gyns) only 66% report that drinking during pregnancy is unsafe. Furthermore, while 82% of Ob/Gyns will inquire about alcohol use of pregnant patients upon their first visit, this inquiry rate falls to 10.6% during subsequent visits [9]. 

Prenatal exposure to ethanol is related to an array of long-term behavioral and cognitive problems. Affected individuals typically have disrupted school experiences, a broad range of cognitive difficulties, decreased social competence and impulsive dyscontrol [10,11]. These problems sometimes, but not always, translate into legal problems or other diagnosable mental disorders [12,13]. Clinical data, as well as animal models of FAE, implicate hippocampal dysfunction as an important feature of these behavioral disorders [14,15]. In rodent models, the early postnatal period (3rd trimester equivalent) has been identified as a critical period for FAE-related hippocampal damage including reduction of neuronal populations in the CA1 region [16,17], impairment of adult hippocampal neurogenesis [18], impaired neural plasticity [15] and disruption of behaviors dependent on an intact hippocampus [19]. 

Imaging studies in patients with non-dysmorphic forms of FASD consistently report anomalies of the corpus callosum, but some also suggest dose-related differences in hippocampal volume and deformities in the head and tail of the hippocampus [20]. Post-mortem studies of the brains of persons affected with FASD are limited in number and confounded by inadequate prenatal history, polydrug abuse, malnutrition and other environmental factors [21]. A broad range of structural anomalies has been reported, but none can be conclusively related to a specific exposure. Furthermore, limited clinical data on low to moderate prenatal alcohol exposure suggest that even light exposure is associated with neurodevelopmental deficits [11]. The current study takes advantage of voluntary and naturalistic drinking patterns [22] in the non-endangered vervet monkey (*Chlorocebus sabeus* St Kitts) to model the type and range of prenatal ethanol exposure frequently reported in epidemiological investigations [2,23]. In addition, this model avoids many of the confounds that plague clinical studies. We previously reported significant neuronal reductions in the frontal cortex and hippocampus in offspring with an average fetal alcohol exposure of 2.5 ± 0.54 g EtOH/Kg/day (range: 1.79–2.98 g EtOH/Kg/day) limited to 4 days a week during the last half of pregnancy [24], as well as reduced stem cell proliferation during infancy [25]. Here we expand on these data to include a larger range of alcohol exposure to investigate the extent of correlations between ethanol exposure variables and hippocampal neuronal populations.

## 2. Materials and Methods

### 2.1. Maternal Ethanol Exposure

As described in greater detail in previous publications [24,26], healthy adult female African green monkeys (*Chlorocebus sabeus*) that voluntarily consumed at least 3 g ethanol/Kg body weight in a 4-h scheduled period were selected for the study. They were housed in harem social groups and examined semi-weekly for early pregnancy. Near the beginning of the third trimester (about day 107, range: 77–118) of the modal 165-day gestation), pregnant females were allowed to drink up to 3.5 g ethanol/Kg body weight (or an isocaloric sucrose control mixture) 4 days per week for a period of 4 h per day. Alcohol was provided in 2-day periods (M, Tu; W, Th) in order to reduce the likelihood of repeated tolerance-withdrawal cycles. Alcohol availability ceased upon parturition so that no infant received additional ethanol in breast milk. The average age of the dam at birth of FAE offspring was 7.25 ± 2.28 years and 6.38 ± 2.61 years for the control group. The total number of offspring for the FAE dams was 7.25 ± 1.25 with 2.5 ± 1.44 offspring being exposed to prenatal alcohol. All dams gained weight during pregnancy, and there was no difference in weight gain between FAE and control dams. Alcohol consumption during pregnancy did not impair the dam’s subsequent fecundity. 

All animals were housed at the Behavioural Sciences Foundation (BSF), St. Kitts in enriched social environments and fed Harlan Teklad high-protein primate chow (5% body weight per day) and fresh local fruit, with water available *ad libitum*. For periods of ethanol consumption, females were trained to move into individual tunnel sections adjacent to their home cages. As a consequence, there was neither sedation nor stress confounding the effects of ethanol consumption. Females were also trained, through reinforcement, to present a leg for phlebotomy so that blood (1 mL, saphenous vein) could be drawn without anesthesia at the end of the drinking period on a single day of weeks 2, 4, 6 and 8 for the estimation of blood ethanol concentration (BEC), using the alcohol dehydrogenase method (Sigma, St. Louis, MO, USA). All protocols were reviewed and approved by the McGill University Animal Care and Use Committee and the BSF Animal Care Committee, both operating under the aegis of the Canadian Council on Animal Care.

### 2.2. Tissue Collection

Offspring were group-housed with their mothers for the first 6 months, then moved to a peer-housing unit as previously described [24]. At about 24 months of age (range 19–24 months), 12 juvenile alcohol-exposed (9 m, 3 f) and 5 juvenile sucrose-control (3 m, 2 f) animals were sacrificed for neuroanatomical evaluation (Table 1). Subjects were sedated with ketamine hydrochloride (10 mg/Kg intramuscular) and sodium pentobarbital (25 mg/Kg, intravenous) using protocols approved by AVMA, then transcardially perfused with PBS, followed by 4% paraformaldehyde in phosphate buffer (pH = 7.4). The brains were removed, blocked into 1 cm slabs, cryoprotected in graded (10–30%) buffered sucrose and frozen at −80 °C, and sectioned (50 µm) in 10 parallel series for morphometry quantification (cresyl-violet) and immunohistochemical analysis according to previously published methods [27,28].

### 2.3. Immunohistochemistry

Systematic sections from one series equally spaced throughout the entire extent of the hippocampus were removed from the vervet brain bank [28] for batch immunohistochemistry processing [29]. Sections were washed 5 times in PBS to remove residual antigen preserve. Doublecortin (DCX), a microtubule-associated phosphoprotein, which is required for neuronal migration and differentiation, has been shown as a putative marker for immature neurons through co-expression studies with the thymidine analogue, 5′-bromo-deoxyuridine (BrdU), which is incorporated into replicating DNA of actively proliferating cells [30,31]. Here, DCX immunohistochemistry was performed according to Rao and Shetty [30]. Briefly, sections were pretreated in 20% methanol, 3% hydrogen peroxide (in 0.1 M PBS) for 30 min at room temperature, washed in 0.1 M PBS, blocked in a 3% normal horse serum (NHS) in PBS, followed by an overnight incubation in goat anti-DCX in the blocking solution at room temperature (1:400; SantaCruz). Following a set of washes, the sections were incubated in biotinylated horse anti-goat IgG (1:200, Vector) for 1.5 h at room temperature. The sections were washed again then incubated in ABC (Vector) for 1.5 h, processed with diaminobenzidine (DAB, Sigma) for visualization and mounted. Sections were dehydrated in graded alcohol solutions, cleared in xylenes and coverslipped with DPX mounting media. As a negative control, a series of sections were processed without the primary antibody and did not produce any staining pattern.

### 2.4. Stereology

Design-based stereological estimation of neuronal numbers was achieved by using the optical fractionator method [32] on cresyl violet-stained sections. The stereological parameters in this study (Table 2) and cyto- and chemo-architecture delineations of the hippocampus were in accordance with Burke et al. [24]. The total estimation of cell numbers (N) was calculated by the following equation:

N = ssf^−1^ × asf^−1^ × tsf^−1^ × ΣQ^−^

where ssf is the section sampling fraction, asf is the sampling fraction, tsf is the thickness-sampling fraction (where the measured thickness of the tissue is divided by the disector height), and ΣQ^−^ is the total number of neurons (defined as having a visible centrally located nucleoli and clearly defined cytoplasm) counted within the disector [24]. A standard ssf of 1/20 and disector volume (x × y × z: 50 × 50 × 10 µm) were used for all subjects.

The disperse and uneven distribution of DCX immunopositive cells in the granular layer of the dentate gyrus would require a large number of disectors to obtain a valid population estimation using the optical disector sampling method described above. Instead, the design-based stereology rare event protocol was used to estimate the total DCX immunopositive cell population with the total estimation of immunopositive cells (N) calculated by the following equation [25]:

N = ssf^−1^ × 1 × 1 × ∑Q^−^

where the area of the counting frame and thickness relative to the sampled area and sampled thickness were the same (such that 100% of the outlined area and thickness were sampled), resulting in asf^−1^ and tsf^−1^ equal to 1. The granular layer of the dentate gyrus was outlined using a 10× objective and the BioQuant software. All cell counts were performed under a 100× oil immersion objective (N.A.1.3). A total of 6–7 sections equally spaced throughout the hippocampus were sampled and each immunopositive cell was counted within the given outlined topography.

### 2.5. Statistical Analysis

A regression analysis was performed using StatView software to determine if a correlation exists between cell counts and daily alcohol ingestion, total alcohol intake, average BEC or the gestational start date of maternal alcohol intake. Control subjects were not included in the regression analysis but are presented in the graph to visually illustrate the comparison between individual FAE subjects to control values. Overall group differences were performed using the non-parametric Mann–Whitney U test (FAE vs. control). The coefficient of error (CE) was calculated as $\sqrt{{\mathrm{meanCE}}^{2}}$. 

## 3. Results

### 3.1. FAE Dose-Dependently Reduced Neuron Number in Vervet CA1, CA2 and CA3

**Figure 1:**
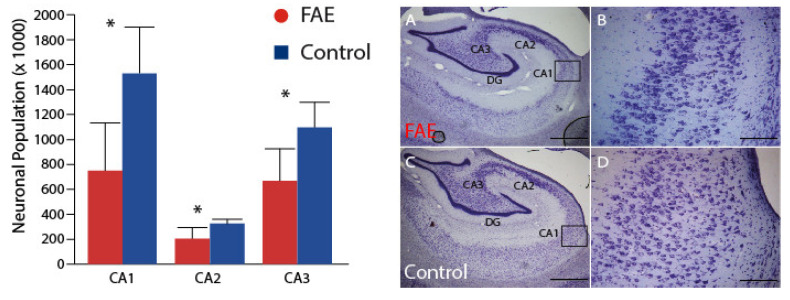
Hippocampal Neuronal Population. FAE subjects, as a group, had significantly lower neuronal populations in hippocampal regions CA1, CA2 and CA3, despite the inclusion of subjects exposed to lower levels of ethanol during gestation. Representative histological sections from FAE (**A**,**B**) and control (**C**,**D**) subjects demonstrate a thinned and sparsely populated pyramidal neuronal layer. * *p* < 0.05 FAE vs. control. (**A**,**C**) scale bar = 1 mm taken at 2.5 magnification; (**B**,**D**) are taken from the CA1 region (inset box) at 10× magnification scale bar = 100 µm.

**Figure 2:**
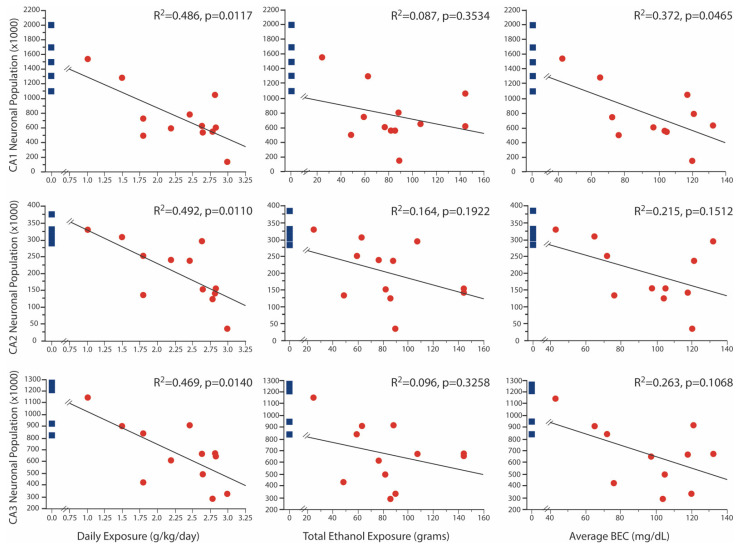
Correlation Between Neuronal Population and FAE. There was a negative correlation between daily ethanol exposure and neuronal populations in all three regions. Neuron numbers in the CA1 region were also negatively correlated with BEC. Control values are shown for each correlation graph for illustration purposes only as they were not incorporated in the correlational analysis.

In a previous publication [24], we reported significant age x treatment effects of FAE on neuronal numbers in the CA1, CA2 and CA3 regions of Ammon’s horn. All of the 2-year-old animals evaluated in that paper contributed to the current analysis; seven 2-year-old animals that had a broader range of fetal ethanol exposure were added. Despite the broader range of exposure, the full sample of FAE offspring still showed significant reductions (Figure 1) of neuron number in CA1 (750 ± 110 *×* 10^3^ vs. 1528 ± 165 *×* 10^3^; *p* < 0.002), CA2 (202 ± 258 *×* 10^3^ vs. 324 ± 140 *×* 10^3^; *p* < 0.005) and CA3 (665 ± 74 *×* 10^3^ vs. 1095 ± 89 *×* 10^3^; *p* = 0.003) as compared to control subjects. Importantly, neuronal populations in all three regions were negatively correlated with daily ethanol exposure, but not with tool exposure or with the duration of ethanol exposure (Figure 2). Additionally, only in the CA1 region was the neuronal population negatively correlated to average BEC, despite the fact that average BEC was significantly correlated with daily ethanol exposure. Visual inspection of these graphs also shows that, for the lowest exposure doses, there was overlap in neuronal counts between FAE and control animals. Correlation analysis of males only yielded similar results with negative correlations between daily exposure and all three regions (CA1 *p* = 0.0084; CA2 *p* = 0.0101; and CA3 *p* = 0.0065), as well as a negative correlation between CA1 neuronal population and average BEC (*p* = 0.0363).

### 3.2. The Volume of CA1 Was Dose-Dependently Lower in FAE Animals 

**Figure 3:**
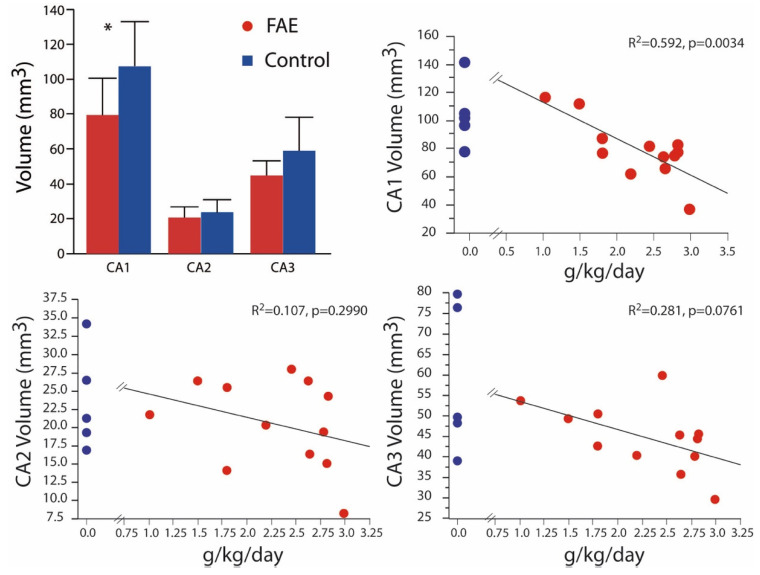
Hippocampal Volume Volumetric differences were isolated to the CA1 with the expanded analysis that includes low FAE-exposed subjects. Volume was negatively correlated with daily exposure in the CA1 region only. Control values are shown for each correlation graph for illustration purposes only as they were not incorporated in the correlational analysis. * *p* < 0.05 FAE vs. control.

In Burke et al. (2015), we also reported significant volumetric differences between FAE and control brains taken from 2-year-old animals for all regions of Ammon’s horn [24]. In the present expanded alcohol exposure sample (Figure 3), significant volumetric differences were observed only in CA1 (79.53 ± 6.06 mm^3^ vs. 107.42 ± 11.42 mm^3^), although mean volumes of CA2 (20.60 ± 1.76 mm^3^ vs. 23.76 ± 3.27 mm^3^) and CA3 (44.84 ± 2.35 mm^3^ vs. 59.00 ± 8.63 mm^3^) were still lower in FAE subjects. Again, there was a significant negative correlation of CA1 volume with daily ethanol intake. Correlation analysis of males only yielded similar results with negative correlations limited to the CA1 region and BEC (*p* = 0.0155) and daily exposure (*p* = 0.0038).

### 3.3. DCX+ Neurons Were Correlated with Daily Dose of Ethanol in FAE Monkeys

**Figure 4:**
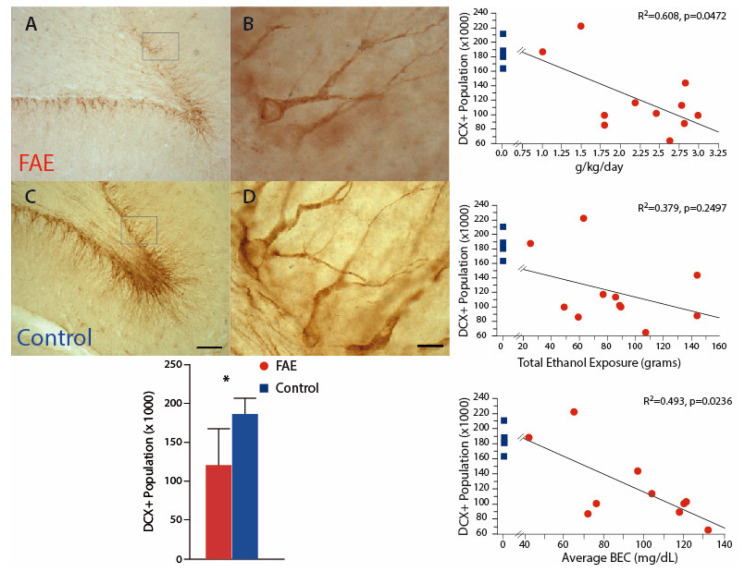
Immature Neuronal Population Inspection of DCX-positive neurons in the dentate gyrus indicates a visibly lower population in FAE (**A**,**B**) versus control (**C**,**D**) subjects. There was a significant reduction of DCX-positive neurons in the dentate gyrus of FAE subjects as compared to controls. The population of DCX-positive neurons was negatively correlated with daily ethanol exposure and average BEC, but not with total exposure. Control values are shown for each correlation graph for illustration purposes only as they were not incorporated in the correlational analysis. * *p* < 0.05 FAE vs. control. (**A**,**C**) scale bar = 100 µm; (**B**,**D**) (inset box) scale bar = 10 µm.

Investigation of developing neurons, using DCX staining, may be relevant both to understanding the dose relationship of ethanol–neuron relationships and to understanding the mechanisms of FAE damage. In this cohort (Figure 4), DCX-immunopositive neurons were significantly decreased by 35.3% in the DG of FAE subjects (120.6 ± 1.41 *×* 10^3^) as compared to control subjects (186.3 ± 10.3 *×* 10^3^; *p* < 0.02). The population of DCX-positive neurons was also negatively correlated with daily ethanol exposure (*r*^2^ = 0.608, *p* < 0.05) and average BEC (*r*^2^ = 0.493, *p* < 0.025), but not with total exposure (*r*^2^ = 0.144, *p* = 0.2497). There was no significant difference between volumes of the dentate gyrus between FAE and control subjects. Correlation analysis of males only yielded similar results with negative correlations between DCX population and BEC (*p* = 0.0164) and daily exposure (*p* = 0.0472).

## 4. Discussion

In this naturalistic model of maternal alcohol consumption, we previously reported significant neuronal loss in the CA1–3 fields following exposures of 2.5 ± 0.54 g/Kg/day. This level of alcohol exposure produced an average blood alcohol content of 98.1 ± 18.6 mg/dL [24] corresponding to levels similar to those found in women after 3–5 standard drinks [33]. Here we expanded the range of daily ethanol exposure (1.01–2.98 g/Kg/day), total ethanol exposure (24.35–143.9 g), and initial day of exposure (E75-118). With the inclusion of subjects exposed gestationally to lower levels of ethanol, the average number of neurons was still significantly lower in all CA subfields (CA1 50.9%, CA2 37.7%, CA3 39.3%). Furthermore, the neuronal population in each CA subfield was negatively correlated to daily ethanol exposure, but not to the embryonic start date or to total ethanol exposure. There was also a negative correlation between the neuronal population in the CA1 and average BEC (Figure 2, Section 3.1). 

We also report here a negative correlation between volumes of CA1 and daily alcohol consumption (Figure 3, Section 3.2). In a previous paper [24], we observed a reduction in all three CA regions of the hippocampus in 2-year-old FAE offspring with ethanol exposure of at least 2 g/Kg/day. Although the average volumes of CA2 and CA3 were lower in this expanded set of exposures, they did not differ significantly from those found in controls. However, the data reported here are consistent with lower neuronal numbers in hippocampi harvested from rodents with higher levels of 3rd trimester ethanol exposure [16,34]. Other rodent studies report that binge-like exposure results in misshapen hippocampi [35] and reduced volumes of CA1 and CA3 [17] as well as a linear negative correlation between brain weight and blood alcohol content [36]. While a clearly defined threshold could not be defined here, in part due to the limitation of animal numbers, overlap between control and FAE values began to be obvious at around 70 mg/dL BEC in the CA1 and appears around 1.5 g ethanol/Kg/day for all subregions (Figure 2, Section 3.1). 

The CA1 region is typically packed with large pyramidal neurons [37], but appears to be particularly vulnerable to FAE, which is consistent with other disorders. Within the hippocampus, there are 122 neuron types that have been identified based on neurotransmitter, morphology, electrophysiology and biomarkers [38]. The specific identity of the vulnerable neurons is beyond the scope of the current study, but there a number of factors that may participate in the developmental vulnerability. The protective neuron–astrocyte–microglia triad are less numerous in the CA1 compared to other hippocampal regions [39]. At a molecular level, the calpain-mediated lysosomal rupture, which is calcium-dependent, may also contribute to accelerated neuronal loss in the CA1 region [40,41,42,43]. Calcium handling and buffering within the CA1 is markedly lower than other hippocampal regions, which is implicated in excitotoxic vulnerability [37,44].

The negative consequences of FAE extend to the immature neuronal population of the dentate gyrus. Here we also report a significant reduction in the FAE subjects, which, like neuronal populations in the CA regions, is negatively correlated to daily ethanol exposure (Figure 4, Section 3.3). The dentate gyrus undergoes substantial postnatal development in non-human primates, with prolonged neurogenesis of granule cells and an estimated 40% of dentate granule cells formed postnatally. Up to 25% of those may be born during the first 3 months of life [45,46]. We have previously shown that stem cell proliferation is significantly reduced shortly after birth in FAE subjects [25], and in both that study and the present communication, we found a significant reduction in DCX-positive cells in the dentate gyrus of FAE offspring. However, by 2 years of age the rate of proliferation in FAE subjects was no different from that in controls [25]. Similar to the neuronal populations, a clear threshold could not be identified, but overlap between control and FAE values ceases at around 70 mg/dL BEC and 1.5 g EtOH/Kg/day. Although a specific threshold of FAE could not be determined, these data add to the growing evidence that low-to-moderate FAE results in compromised hippocampal functioning [47,48] affecting synaptic activity [47], connectivity [47,49], neurogenesis [50], and plasticity [51,52] with adverse learning and behavior [53,54,55].

In both clinical and animal-model studies, the effects of more moderate and light prenatal ethanol exposure are not well delineated in part due to the inconsistent definitions of low-to-moderate drinking, variable maternal consumption patterns, and methodological issues related to maternal self-reporting [56,57]. For example, using the Behaviour Rating Inventory of Executive Function (BRIEF) parent/teacher rating survey, Skobergo et al. [58] report that low to moderate maternal drinking, as defined as one to four (low) or five to eight (moderate) drinks per week does not affect executive functioning at 5 years of age. Also, in an adolescent longitudinal study, a low level of maternal alcohol consumption (<1 glass ethanol/day during pregnancy) was not associated with attention or learning outcomes, but moderate to high exposure (>1 glass ethanol/day during pregnancy) was associated with learning difficulties as assessed by the Child Behavior Checklist [59]. Academic achievement in children (aged 8–9 years) was found not to be associated with low (1–2 standard drinks per drinking occasion and <7 per week) or moderate (3–4 standard drinks/occasion and <7 per week) prenatal alcohol exposure [60]. However, in the last decade, multiple investigations have reported problems of behavior, cognition and attention following alcohol exposure throughout pregnancy, even at low doses of <1 average daily drinks [61]. Neurobehavioural outcomes in non-human primates that were exposed to moderate fetal alcohol exposure (maternal alcohol consumption of 0.6 gEtOH/Kg/day yielding a blood alcohol concentration of 20–50 mg/dL) parallel clinical assessments affecting motor, attention, orientation, sensory, irritability and emotional domains potentially affecting performance on cognitive, executive, learning and memory tasks [62,63]. 

With respect to socioemotional issues, an early study [64] using the Child Behavior Checklist reported that drinking even very low amounts of ethanol during pregnancy was associated with aggressive and delinquent behavior (externalizing behavior) at 6–7 years of age, while drinking more moderate levels of ethanol was associated with anxious, depressed and withdrawn behavior (internalizing behavior). Furthermore, any prenatal ethanol exposure was correlated with delinquent behaviour during early childhood [64]. A more recent prospective study [65] using the same assessment instrument argues that low (2–6 drinks/week) and moderate (7–10 drinks/week) levels of maternal drinking during pregnancy were associated with a reduction in internalizing and externalizing behaviour problems in adolescents compared to non-FAE-exposed subjects. Particularly relevant to the findings reported here are reports of changes in brain structure [66] and synaptic plasticity [51] following low-to-moderate prenatal ethanol exposure. Despite the preponderance of the evidence that prenatal ethanol is toxic, methodological problems of defining exposure [67] and the questionable validity of maternal self-reports [68] limit the certainty of clinical studies and necessitate controlled animal studies such as the one reported here.

Although a discussion of the molecular underpinnings of neuronal deficits following fetal ethanol exposure is beyond the scope of this paper, it may nonetheless be relevant to reflect on certain aspects of impaired postnatal neurogenesis as this might provide a potential target for neurotherapy, either directly or indirectly. While the paucity of DCX-labelled cells in the dentate gyrus cells might argue for a simple toxicity model [69], toxicity is clearly incomplete, even in models with a much higher ethanol exposure than the one described here [18,70,71]. Another possibility is that prenatal ethanol exposure either delays neurogenesis and/or migration of granule cells. The extent of the neurogenic capacity in the dentate gyrus following prenatal ethanol exposure in rodent models remains a contested topic [50,70,72], but multiple studies with different levels and patterns of prenatal alcohol exposure report behavioral improvement and increased levels of DCX+ cells in dentate gyrus in FAE animals housed in enriched environments [73,74,75].

A final issue that merits discussion is the finding that the mean daily dose of ethanol was a stronger predictor of neuronal number than either the exact timing of the initiation of ethanol administration or the measured blood ethanol concentration. Ethanol exposure in this study was targeted to the last trimester before delivery, but about half of the animals studied here began alcohol exposure slightly before the beginning of the third trimester (day 110 of the modal 165-day gestation period). Also, although both BEC (*r*^2^ = 0.818) and total alcohol consumption (*r*^2^ = 0.673) were significantly correlated with average daily alcohol consumption (g/Kg/day), these measures are not identical and not equally robust. In some individual animals, daily consumption varied by as much as 38%. In addition, collection of blood for the measurement of BEC occurred intermittently, and it could not be ensured that every animal had consumed the normative amount of ethanol on the day of blood collection. Moreover, BEC was measured in blood taken at the end of the drinking period, and how this relates to peak BEC (which is suggested to be a more salient dose metric for ethanol-induced brain toxicity [76]), is not known. In the clinical context, this would be consistent with the notion that those pregnant females that drank consistently day after day would be more likely to have impaired infants than those with more intermittent consumption [57]. 

## 5. Conclusions

Despite public health advisories against the consumption of any level of beverage ethanol during pregnancy, controversies regarding a possible “safe level” of drinking continue to abound. As reviewed above, it may not be possible to gather clinical evidence adequate to resolve this issue. In a naturalistic non-human primate model of FAE, we are able to control for many of these confounds. Using the sensitive and robust methodologies of cell counting and stereology, we provide evidence of significant dose-responsive deficits in neuron number and regional volumes of hippocampal structures. A similar decrease was observed with DCX-positive developing cells in the dentate gyrus. This study does not specifically identify a safe level of FAE below which the hippocampus is protected but does provide evidence that above the dose of 1.5 g/Kg ethanol (equivalent to a blood ethanol concentration of 70 mg/dL) there is no overlap between neuronal counts in control animals and those exposed to ethanol during pregnancy. An important limitation is that this study evaluates only three measurements: volumes and neuronal populations in CA fields and immature dentate neurons. Other important types of damage, such as impaired synaptic plasticity, altered firing patterns or disrupted circuitry may occur at lower doses. It is evident that low-to-moderate fetal alcohol exposure affects multiple domains of neural function and behaviour, but it is clear that from clinical and animal studies that low-to-moderate prenatal ethanol exposure warrants further investigation. 

## Figures and Tables

**Table 1:** Subject Profile of animals exposed to ethanol in utero. The period of exposure was calculated retrospectively from date of birth. Total ethanol exposure and average exposure are based on the amount the dam drank during pregnancy and her body weight before pregnancy. Blood ethanol content (BEC) was measured 3–4 times during the alcohol exposure period for each dam and averaged.

Animal	Sex	Alc or Suc Started *	Average Alc (g/Kg/day)	Total Alc Exposure (g)	Average BEC	Age at Sacrifice
O2762-5	m	117	1.01	24.35	43	24 mos
O5066-1	m	109	1.49	63.9	65	24 mos
O5106-1	m	109	1.79	59.2	72	24 mos
O5332-1	f	106	2.19	76.5	91	24 mos
O3082-3	f	115	1.79	48.3	76	24 mos
O5219-1	m	103	2.45	88.3	121	22 mos
O3295-3	m	95	2.63	107.0	132	21 mos
O5011-3	m	77	2.81	135.1	118	24 mos
O3295-2	m	75	2.82	143.9	97	24 mos
O3307-2	f	111	2.64	81.8	105	24 mos
O3327-2	m	113	2.77	85.73	104	22 mos
O3327-1	m	115	2.98	89.4	120	21 mos
O5603-2	m	--	0	sucrose		22 mos
O5151-3	m	--	0	sucrose		19 mos
O6036-1	f	--	0	sucrose		24 mos
O4056-3	f	--	0	sucrose		24 mos
O3060-5	m	--	0	sucrose		21 mos

* Day of gestation alcohol or sucrose treatement started.

**Table 2:** Stereology Parameters from unilateral cerebral cortex neuron and volume estimation of control and fetal alcohol-exposed (FAE) monkeys (ΣQ^−^ total number of neurons sampled; ΣF number of disectors sampled; V cortical gray volume; N total number of neurons CE (ΣQ^−^) coefficient of error for ΣQ; CE (ΣF) coefficient of error of ΣF; and CE (N) coefficient of error for N). Values are given as mean (CV = SD/mean).

Subregion	Average Number of Sections	Average Tissue Thickness (µm)	Mean ∑F	Mean ∑Q^−^	Average x − y Step	Mean V^ref^ (mm^3^)	Mean N (In Millions)	Mean CE (N) *
***Control***								
CA1	13.4	15.81	118	104	1026	1073	1.528	0.089
CA2	13.4	15.94	97	108	479	237	0.324	0.126
CA3	13.6	15.57	110	171	756	590	1.095	0.099
***FAE***								
CA1	12.8	16.88	162	122	698	795	0.750	0.096
CA2	12.8	17.75	160	116	351	206	0.202	0.113
CA3	12.9	16.66	157	185	526	448	0.665	0.115

* Mean CE is calculated $\sqrt{{\mathrm{meanCE}}^{2}}$.

## Data Availability

Not applicable.
